# Spin-canted Mn–Mn coupling in symmetry-broken metal chloride dimer with dual-responsive luminescence and sensing

**DOI:** 10.1038/s41377-025-02154-9

**Published:** 2026-01-28

**Authors:** Guojun Zhou, Pei Wang, Qiqiong Ren, Nan Zhang, Jin Lv, Yilin Mao, Jianwei Qiao, Xian-Ming Zhang

**Affiliations:** 1 Key Laboratory of Magnetic Molecules and Magnetic Information Material, Ministry of Education, School of Chemistry and Chemistry Engineering, Shanxi Normal University, Taiyuan, China; 2 College of Chemistry & Chemical Engineering, College of Physics and Optoelectronics, Taiyuan University of Technology, Taiyuan, China; 3 Department of Applied Chemistry, Yuncheng University, Yuncheng, Shanxi China

**Keywords:** Micro-optics, Photonic crystals

## Abstract

Magneto-optical coupling provides a powerful alternative to crystal field engineering for modulating Mn^2+^ luminescence. However, precise control over Mn–Mn coupling is hindered by the complex spin-electron super-exchange interactions. Herein, we report a symmetry-broken Mn(II) chloride dimer, (C_10_H_20_O_5_Mn)(CH_3_CN)MnCl_4_, synthesized through a crown-ether-assisted supramolecular strategy. The dimer features a 7-coordinated pentagonal bipyramid and a 4-coordinated tetrahedron linked by a distorted Mn–Cl–Mn bridge (129°), which promotes rare spin-canted Mn–Mn coupling and creates a novel Mn–Mn luminescent center. This center exhibits a red emission at 638 nm with an unusually short lifetime of 0.42 ms, which is attributed to the relaxation of spin-forbidden *d*–*d* transitions. Notably, the emission undergoes a 30 nm blue-shift upon heating (5–305 K) due to the thermal suppression of spin-canting, and a 40 nm blue-shift under applied pressure (0–20 MPa) resulting from reduced orbital overlap. This dual-responsive luminescence originates from spin-canted weak ferromagnetism, which induces a rearrangement of energy-levels by separating antibonding orbitals. Using this effect, we have demonstrated an optical manometer for real-time underwater depth sensing. These findings highlight spin-canted Mn(II) dimers as a promising platform for stimuli-responsive luminescence and reveal a new mechanism for *d–d* transition modulation.

## Abstract (web summary)

A symmetry-broken Mn(II) dimer enables the dual-stimuli-responsive luminescence through spin-canted Mn**‒**Mn coupling, providing a new mechanism for modulating *d*-*d* transitions and serving an optical manometer for sensing.

## Graphical abstract

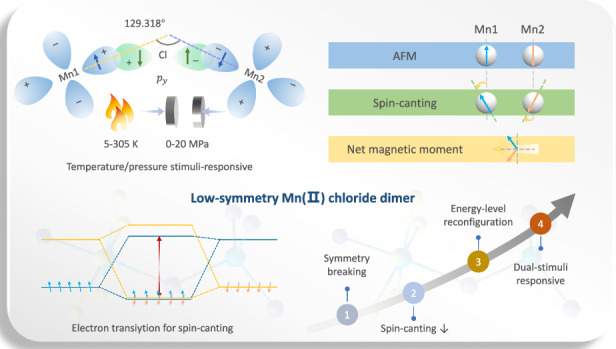

## Introduction

Stimuli-responsive luminescent materials have emerged as a versatile platform for advanced optoelectronic applications, especially in precision sensing, biomechanical imaging, and next-generation human-machine interfaces, due to their exceptional sensitivity, ultrafast response dynamics, and unique capability for real-time spatial information acquisition^1–7^. Recently, organic-inorganic hybrid Mn^2+^-based metal halides have garnered significant interest as a promising class for constructing such functional systems, benefiting from their environmental sustainability, cost-effectiveness, and distinctive photophysical properties^8–14^. The characteristic *d-d* transition emission (^4^T_1_ → ^6^A_1_) in Mn^2+^ systems is governed by crystal field strength^15–20^. Current tuning strategies primarily rely on the chemical modification of coordination environments or crystal field modulation via Jahn-Teller distortions, yet they encounter challenges such as complex regulation processes, narrow dynamic ranges, and limited stimulus tunability^21–25^.

**Scheme 1:**
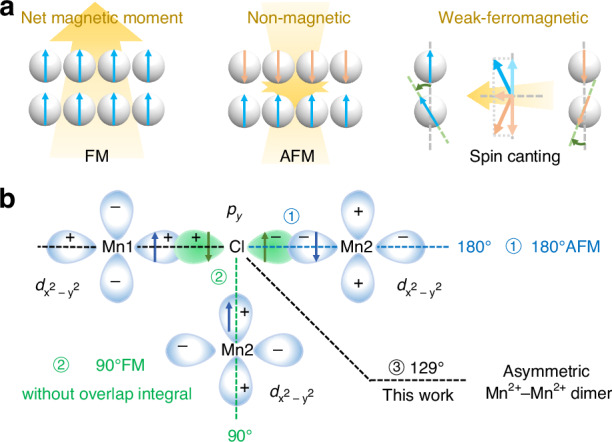
Mn‒Mn coupling interactions. **a** The magnetic moment arrangements of antiferromagnetic (AFM), ferromagnetic (FM) and spin-canting. **b** The orbital overlap scheme for Mn‒Cl‒Mn super-exchange pathways at characteristic bond angles: 90°, 180° and 129° (this work)

Magneto-optical coupling provides a powerful approach for tailoring Mn^2+^ photoluminescence (PL) by manipulating its spin configuration^26–28^. Super-exchange interactions govern the spin arrangement of Mn^2+^ ions, leading to their spontaneous magnetic order^29,30^. These magnetic ordering structures selectively modulate the energy levels and *d-d* transitions of Mn^2+^, leading to distinct PL behaviors in magnetically ordered states that differ from crystal field-dominated emissions in paramagnetic phases^31–34^. Studies have demonstrated that antiferromagnetic/ferromagnetic (AFM/FM) coupling induces contrasting spectral shifts (blue/red) via differential *d*-orbital splitting^35^. For instance, controllable near-infrared emission in ABF_3_ fluoride perovskites was achieved by tuning the Mn‒F‒Mn bond angles and lengths via the FM super-exchange across effective dimers^36^. Li et al. reported an unusual red emission at 620 nm in a tetra-coordinated lattice, which was attributed to the Mn‒Mn magnetic coupling interaction^37^. In addition, it was demonstrated by Ye et al. through variable-temperature PL and magneto-spectroscopy that the antiparallel spin arrangement in 1D Mn^2+^ chains suppresses energy migration, thereby enhancing the photoluminescence quantum yield (PLQY)^38^. Despite these advances, research remains predominantly focused on collinear spin orders (AFM/FM) in high-symmetry architectures, frequently relying on doping strategies. Furthermore, this doping approach often leads to poorly controlled mixtures of isolated ions, dimers, and multimers, which obscures the precise structure-property relationships^39^. So far, the electron transition mechanisms in AFM/FM configurations are well-established, whereas the role of spin-canted Mn‒Mn coupling interactions in symmetry-broken systems remains unexplored due to synthetic challenges in distortion-controlled assembly. Scheme 1 summarizes the magnetic coupling modes with an emphasis on the influence of bond angle on spin arrangements. As illustrated in Scheme 1a, the magnetic moments adopt distinct arrangements in FM, AFM, and spin-canted configurations. Scheme 1b depicts representative M–X–M bonding geometries where the bond angle of 180° favors AFM arrangement, whereas the bond angle of 90° promotes FM coupling. A spin-canted configuration emerges at 129°, allowing for symmetry breaking. It is recognized that the Mn–X–Mn bond angle is a key parameter determining the magnetic coupling mode. The spin-canted configuration emerging at intermediate angles provides a potential pathway for dynamic modulation under external stimuli.

In this work, we report the synthesis of Mn(II) chloride dimer through a crown-ether-assisted supramolecular approach. The resulting (C_10_H_20_O_5_Mn)(CH_3_CN)MnCl_4_ features an unprecedented symmetry-broken coordination geometry that combines a 7-coordinated pentagonal bipyramid and a 4-coordinated tetrahedron, linked by a Mn–Cl–Mn bridge with a bond angle of 129°. This distinctive structure facilitates spin-canted super-exchange interactions, which establishes a novel luminescent center of Mn‒Mn dimers, resulting in red emission at 638 nm with a short lifetime of 0.42 ms. The weak ferromagnetic interaction resulting from spin-canted Mn–Mn coupling induces a reconfiguration of the energy levels, which facilitates a distinct *d*-*d* transition mechanism that operates differently from conventional AFM/FM frameworks. Not only that, the system exhibits dual responsiveness to both pressure and temperature, which is manifested through blue-shifted emission resulting from suppressed orbital overlap and modulated spin-canting. Capitalizing on its pressure sensitivity, we demonstrate an optical underwater manometer for real-time depth monitoring. This work not only elucidates a novel *d-d* transition mechanism by symmetry-broken coordination, but also highlights magnetic coupling interactions as a versatile design strategy for developing stimuli-responsive luminescent materials.

## Results

**Fig. 1:**
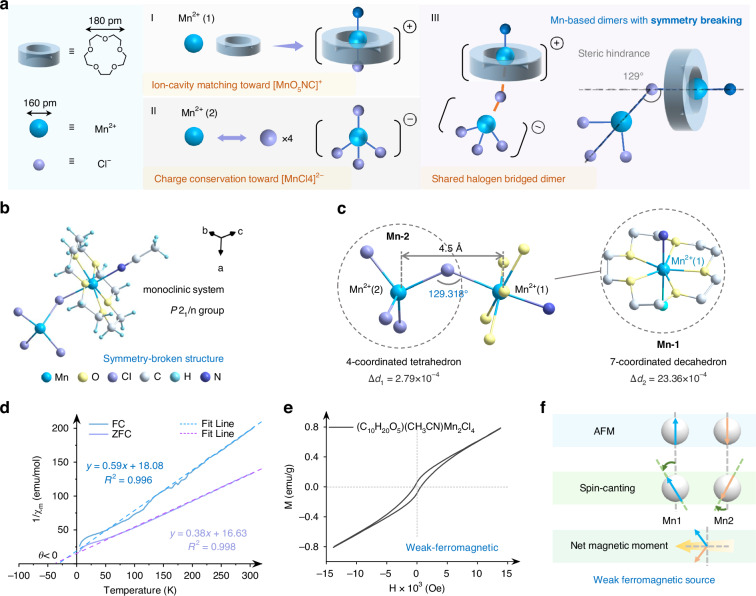
**Crystal structures and magnetic properties**. **a** Schematic diagram of the supramolecular self-assembly process of (C_10_H_20_O_5_Mn)(CH_3_CN)MnCl_4_. **b** Asymmetric units of (C_10_H_20_O_5_Mn)(CH_3_CN)MnCl_4_. **c** Structure distortion and highlighting the Mn···Mn distance and Mn‒Cl‒Mn bond angle with omitted C/H atoms to emphasize the inorganic framework. **d** Temperature-dependent magnetic susceptibility curves under zero-field-cooled (ZFC) and field-cooled (FC) modes. Curie-Weiss fittings of the plot of *χ*_m_^−1^ vs T from 50 K to 305 K. **e** Field-dependent magnetization measured at room temperature. The vertical axis represents magnetization intensity (M) and the horizontal axis represents magnetic field (H). **f** Schematic illustration of magnetic moment distribution of AFM ordering and spin-canting configurations

The centimeter-sized single-crystal of (C_10_H_20_O_5_Mn)(CH_3_CN)MnCl_4_ was synthesized using a straightforward cooling crystallization method, as illustrated in Fig. S1. It crystallizes in the low-symmetry monoclinic *P*2_1_/n space group and displays a unique isolated zero-dimensional (0D) host-guest structure (Fig. S2). The crystallographic asymmetric unit of (C_10_H_20_O_5_Mn)(CH_3_CN)MnCl_4_ consists of a chloride-bridged dimer incorporating two distinct manganese coordination centers (**Mn-1** and **Mn-2**), as depicted in Fig. 1b. As shown in Fig. 1a, the cavity diameter of 15-crown-5 (180 pm) exhibits an excellent size match with the Mn^2+^ ion (166 pm), facilitating optimal host-guest interactions in **Mn-1**^40^. The Mn^2+^ ion establishes strong electrostatic bonds with the five oxygen atoms of 15-crown-5, while simultaneously coordinating to one nitrogen atom and one chloride ion, resulting in a well-defined 7-coordinated pentagonal bipyramidal unit (**Mn-1**). To maintain charge neutrality, a second Mn^2+^ ion coordinates with four chloride anions to form a tetrahedral unit (**Mn-2**). The asymmetric dimer features a relatively short Mn···Mn distance of 4.5 Å, with the bridging chloride ions forming Mn‒Cl‒Mn angles of 129° (Fig. 1c). The short Mn···Mn distance implies the existence of super-exchange interactions between two magnetic manganese ions^41,42^. Notably, the Mn‒Cl‒Mn bond angle in (C_10_H_20_O_5_Mn)(CH_3_CN)MnCl_4_ is measured as 129°, which lies between the ideal angles of 180° (for AFM coupling) and 90° (for FM coupling). This intermediate value thus suggests a non-collinear spin arrangement, thereby distinguishing from conventional FM or AFM configurations^43–46^. The electron spins are no longer arranged in a strictly parallel or antiparallel configuration, but allow for a certain degree of spin-canting. Furthermore, the distortion degrees of the **Mn-1** and **Mn-2** polyhedra were evaluated using the M$-$X bond length distortion (∆*d*) formula^47,48^:

1 $$\Delta d=\frac{1}{7}\mathop{\sum }\limits_{n=1}^{7}{\left(\frac{{d}_{n}-\bar{d}}{\bar{d}}\right)}^{2}\left(\mathrm{Seven\; coordination}\right)$$

2 $$\Delta d=\frac{1}{4}\mathop{\sum }\limits_{n=1}^{4}{\left(\frac{{d}_{n}-\bar{d}}{\bar{d}}\right)}^{2}\left(\mathrm{Four\; coordination}\right)$$

where *d*_n_ represents the individual M$-$X (X = Cl, O, N) bond lengths, and $\bar{d}$ is the average bond length. The values of ∆*d* for **Mn-1** and **Mn-2** are 23.36 × 10^**−**4^ and 2.79 × 10^**−**4^, respectively. The intrinsic asymmetric coordination of (C_10_H_20_O_5_Mn)(CH_3_CN) MnCl_4_ coupled with enhanced geometric distortions, induces an asymmetric crystal field. This promotes the breaking of structural symmetry and creates favorable conditions for spin-canting. The crystallographic information file (CIF) has been deposited at the Cambridge Crystallographic Data Centre (CCDC 2489969). Detailed structural data, including the atomic coordinates, displacement parameters, and main bond lengths, bond angles and hydrogen bonds, are listed in Tables S1–S5, respectively. X-ray photoelectron spectroscopy (XPS) and scanning electron microscopy (SEM) (Figs. S3–S4) were employed to further confirm the elemental compositions and chemical valence states of (C_10_H_20_O_5_)(CH_3_CN)Mn_2_Cl_4_.

To validate the super-exchange interactions in the asymmetric dimers, we performed the detailed magnetic characterization of (C_10_H_20_O_5_Mn)(CH_3_CN)MnCl_4_ using a Physical Property Measurement System (PPMS). Temperature-dependent magnetization measurements were carried out from 2 to 305 K under an applied field of 10 kOe, as shown in Fig. 1d. The magnetic susceptibility data were analyzed based on the Curie-Weiss law^49^:

3 $${\chi }_{m}=\frac{C}{T-\theta }$$

where *θ* denotes the Weiss temperature, *T* represents the testing temperature, *C* is the Curie constant, and ${\chi }_{m}$ is the molar susceptibility. The values of *θ* obtained under zero-field-cooled (ZFC) and field-cooled (FC) conditions were measured to be −43.46 K and −30.64 K. The negative Weiss constant (*θ* < 0) indicates dominant AFM coupling between adjacent Mn^2+^ ions. However, the distinct divergence between ZFC and FC magnetization curves implies the presence of a non-collinear spin arrangement, i.e., spin-canting, which results in a weak ferromagnetic component. The hysteresis loop measured by Vibrating Sample Magnetometer (VSM) at room temperature (Fig. 1e) confirms the weak ferromagnetism in (C_10_H_20_O_5_Mn)(CH_3_CN)MnCl_4_. These collective magnetic properties provide strong evidence for the occurrence of spin-canting in (C_10_H_20_O_5_Mn)(CH_3_CN)MnCl_4_, which can be attributed to two principal structural factors: (1) the nonstandard Mn‒Cl‒Mn bond angle of 129° and (2) the distinct crystal-field environments within the asymmetric dimer unit. Fig. 1f schematically illustrates the magnetic moment distribution for both AFM and spin-canting configurations. The antiparallel arrangement of magnetic moments, combined with a canting angle, results in a small net magnetic moment, accounting for the weak spontaneous magnetization in this system.

**Fig. 2:**
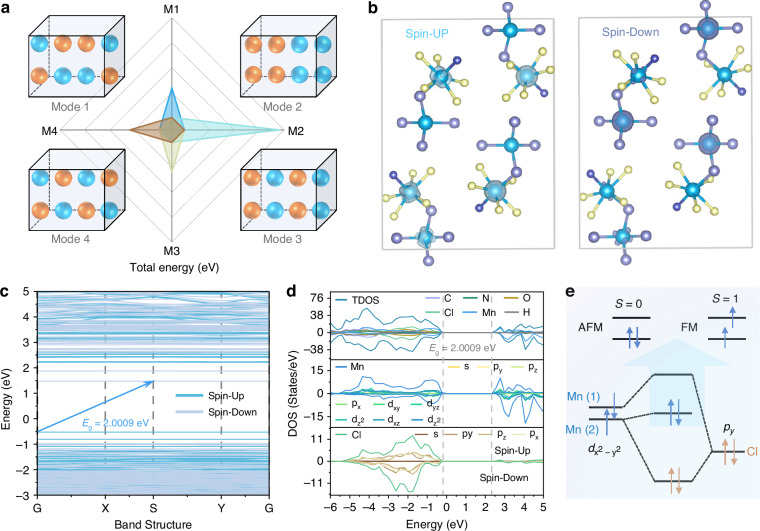
**Density functional theory (DFT) theoretical calculations**. **a** Magnetic structures including three different AFM magnetic models and one FM magnetic model along with the total energy of diverse hypothetical models for Mn‒Mn coupling. The blue and orange balls represent the spin-up and spin-down, respectively. **b** Isosurface plots of the electron spin density of (C_10_H_20_O_5_Mn)(CH_3_CN)MnCl_4_. The yellow and purple areas represent the spin-up and spin-down, respectively. **c** The calculated electronic band structures of (C_10_H_20_O_5_Mn)(CH_3_CN)MnCl_4_. **d** The total and partial density of states of (C_10_H_20_O_5_Mn)(CH_3_CN)MnCl_4_. **e** schematic diagram of molecular orbitals of (C_10_H_20_O_5_Mn)(CH_3_CN)MnCl_4_

As illustrated in Fig. S5, we systematically evaluated three distinct AFM coupling models and one FM coupling model for (C_10_H_20_O_5_Mn)(CH_3_CN)MnCl_4_, with fixed magnetic moments for each Mn^2+^ ion. The total energies of these models were calculated by density functional theory (DFT) to obtain the most stable magnetic model. Detailed energy values for each model are provided in Table S6. Notably, the total energies of Models 1, 3, and 4 were relatively lower than that of Model 2. Furthermore, their close total energies indicate the absence of super-exchange channels between adjacent dimers, which can be attributed to the large Mn···Mn distance (>5.6 Å, Fig. S6) between the dimers. Consequently, the super-exchange interactions are confined to between 7-coordinated pentagonal bipyramid (**Mn-1**) and 4-coordinated tetrahedra (**Mn-2**) within each dimer. As illustrated in Fig. 2a, the antiparallel arrangements of magnetic moments correspond to the most stable configuration. Therefore, Model 3 with the lowest total energy (*E*_M3_ = −1066.0161 eV) was selected as the research object. Subsequently, the three-dimensional spatial distributions of both the charge density and spin density for (C_10_H_20_O_5_Mn)(CH_3_CN)MnCl_4_ under spin-orbit coupling (SOC) were further derived. As shown in Fig. S7 and Fig. 2b, the charge density is primarily localized on the oxygen (O) and manganese (Mn) atoms, whereas the unpaired electrons are exclusively distributed over the Mn^2+^ ions. Specifically, the spin-up electrons are principally concentrated on the Mn^2+^ (1) ions, while the Mn^2+^ (2) ions are predominantly occupied by spin-down electrons. Certain Mn^2+^ ions exhibit concurrent distributions of both spin-up and spin-down electrons, further supporting the occurrence of spin-canting in the magnetic moments of the Mn^2+^ ions.

**Fig. 3:**
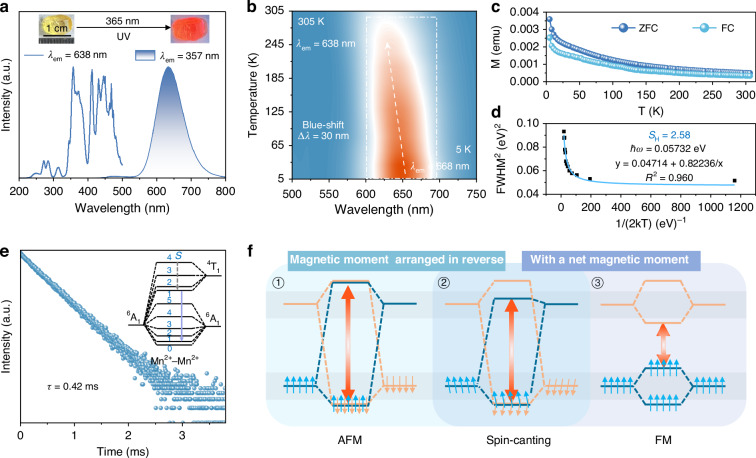
**Optical properties and mechanistic analysis.** **a** PLE and PL spectra of (C_10_H_20_O_5_Mn)(CH_3_CN)MnCl_4_ under 357 nm ultraviolet (UV) excitation. The inset depicts the single-crystal photographs under natural light and UV lamp. **b** Temperature-dependent PL spectra under 357 nm UV excitation from 5 K to 305 K. **c** Temperature-dependent magnetization intensity (M) under ZFC and FC modes. **d** The trend of FWHM^2^ with temperature and the fitting line of the Huang-Rhys factor (S_H_). **e** PL decay curves of (C_10_H_20_O_5_Mn)(CH_3_CN)MnCl_4_ under 357 nm excitation at room temperature. The inset illustrates a schematic representation of the formation of new luminescent centers by Mn‒Mn coupling interaction. The vertical purple solid line shows the spin-allowed transition (Δ*S* = 0). **f** Schematic diagrams of electron *d-d* transition in Mn‒Mn coupling interaction, including AFM, spin-canting and FM

As illustrated in Fig. 3a, (C_10_H_20_O_5_Mn)(CH_3_CN)MnCl_4_ exhibits a bright red emission at 638 nm under 357 nm ultraviolet (UV) excitation. Temperature-dependent PL spectra in Fig. 3b reveal only a single emission peak even at temperatures as low as 5 K, which eliminates the possibility of energy transfer between Mn^2+^ (2) and Mn^2+^ (1). The fluorescence decay curve can be accurately fitted by a single-exponential decay equation (Fig. 3e)^50–52^:

4 $$I\left(t\right)=A\exp \left(-\frac{t}{\tau }\right)$$

where *I*(t) denotes the PL intensity at time *t*, *A* is a constant, and *τ* is the decay time for an exponential component. The fluorescence lifetime was determined to be 0.42 ms, which is considerably shorter than those of most conventional manganese chlorides (Table S7). This distinct photophysical behavior originates from two synergistic effects: (1) the formation of a new luminescent center via Mn‒Mn super-exchange interactions within the dimeric^31^, and (2) the symmetry breaking induced by the asymmetric coordination geometry and spin-canting, which relaxes the spin-selection rules and consequently shortens the fluorescence lifetime^53^. To elucidate the electronic transition mechanism, DFT calculations were conducted to determine the electronic band structure of (C_10_H_20_O_5_Mn)(CH_3_CN)MnCl_4_. The results reveal an indirect bandgap of 2.00 eV, with the valence band maximum (VBM) and conduction band minimum (CBM) dominated by Mn^2+^ and Cl^‒^ orbitals, as shown in Fig. 2c, d. While the Cl^‒^ ions primarily serve structural stabilization roles without significant involvement in magnetic coupling (Fig. 2e), we only discuss the arrangement and transition of electrons localized in Mn‒Mn ion pairs. According to the Heisenberg model, the spin-canting modifies the system’s effective spin quantum number (*S*), consequently altering both energy level spacing and center of gravity, which fundamentally perturbs the transition selection rules^54^. As shown in Fig. 3f, we established a theoretical framework by extending conventional electron transition models for FM and AFM systems to account for the unique spin-canting configuration in this system. The spin-up and spin-down states of *d* electrons are marked in blue and orange, respectively. The occupied spin-up *d* state of one Mn^2+^ ion interacts with the unoccupied spin-up *d* state of another Mn^2+^ ion, resulting in the formation of an occupied bonding orbital and an unoccupied antibonding orbital. The weak ferromagnetism modifies the energy level arrangement in (C_10_H_20_O_5_Mn)(CH_3_CN)MnCl_4_, leading to the non-overlap of antibonding orbitals. Consequently, the energy gap for electron transitions from the ground state to the lowest excited state is situated between those of AFM and FM configurations.

As the temperature increases from 5 to 305 K, the temperature-dependent PL spectra exhibit a continuous blue shift, with the emission peak shifting from 668 nm to 638 nm, accompanied by a broadening of the full width at half maximum (FWHM) (Figs. 3b, S8). The temperature-dependent FWHM variation in the 5-305 K range is well fitted by the following equation^55,56^:

5 $$F{\rm{W}}{HM}=2.36\sqrt{{S}_{H}}\hslash {{\rm{\omega }}}_{\mathrm{phonon}}\sqrt{\coth \frac{\hslash {{\rm{\omega }}}_{\mathrm{phonon}}}{{2k}_{B}T}}$$

Further simplified as:

6 $$F{\rm{W}}{{HM}}^{2}=a+\frac{b}{\frac{1}{2{kT}}}$$

where *a* = 5.57 × *S*_H_ × ($\hslash$*ω*_phonon_)^2^ and *b* = 5.57 × *S*_H_ × ($\hslash$*ω*_phonon_), *ħ, ω*_phonon_, *k*_B_ and *S*_H_ represent the Planck constant, phonon frequency, Boltzmann’s constant and Huang-Rhys factor, respectively. As shown in Fig. 3d, the calculated Huang-Rhys factor of 2.58 implies a relatively rigid lattice and weak electron-phonon coupling in (C_10_H_20_O_5_Mn)(CH_3_CN)MnCl_4_, thereby ruling out the possibility that the observed blue shift in emission wavelength from 668 to 638 nm originates from the temperature-induced lattice expansion. By contrast, the temperature-dependent magnetization curves reveal a gradual decrease in magnetic intensity with increasing temperature for (C_10_H_20_O_5_Mn)(CH_3_CN)MnCl_4_, as illustrated in Fig. 3c. The thermally suppressed spin-canting reduces the orbital separation, which directly accounts for the PL blue shift. These findings confirm that the magnetic moment arrangement can be modulated by temperature, with spin-canting acting as a crucial mediator in the temperature-dependent Magneto-optical coupling.

**Fig. 4:**
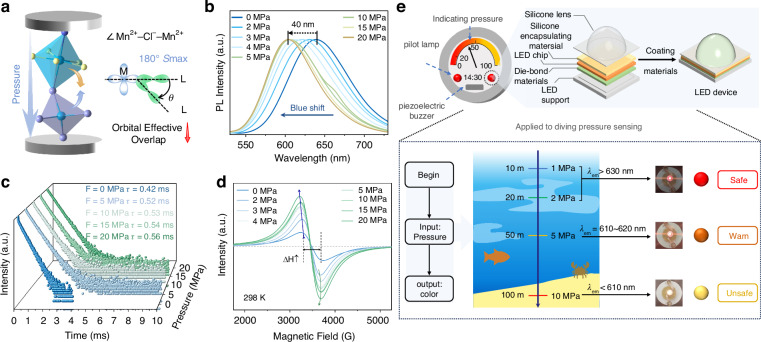
**Spin-canting-mediated pressure response and underwater manometer application**. **a** Schematic illustration of the pressure application process and pressure-induced variational orbital overlap. **b** Pressure-dependent PL spectra with the pressure range of 0-20 MPa. **c** PL decay curves upon 357 nm excitation under varying pressures from 0 to 20 MPa, along with fluorescence lifetimes increasing. **d** EPR spectra showing pressure-dependent linewidth broadening (0-20 MPa) at 298 K. **e** The schematic diagram of the underwater manometer and its application scenarios achieved underwater pressure monitoring at 2 MPa, 5 MPa and 10 MPa, along with the corresponding fluorescence color changes from red to orange to yellow

In addition to its temperature sensitivity, (C_10_H_20_O_5_Mn)(CH_3_CN)MnCl_4_ also exhibits the pressure-responsive luminescence behavior. As illustrated in Figs. 4b, S9, as the pressure increases from 0 to 20 MPa, the PL spectra display a significant blue-shift of around 40 nm, accompanied by a continuous increasing in fluorescence lifetime (Fig. 4c). The emergence of super-exchange interactions between adjacent Mn^2+^ ions is characterized by the collapse of hyperfine splitting features and a significant broadening in linewidth (ΔH) in the electron paramagnetic resonance (EPR) spectrum^57,58^. As shown in Fig. 4d, the EPR spectra display a single broad peak, in contrast to the characteristic six-line hyperfine splitting of Mn^2+^, which can be attributed to the strong spin relaxation effects in (C_10_H_20_O_5_Mn)(CH_3_CN)MnCl_4_. Pressure-dependent studies reveal two distinct regimes: (1) below 10 MPa, the EPR linewidth shows progressive broadening, and (2) above 10 MPa, the EPR linewidth almost saturates to a constant value (Fig. 4d, Table S8). These observations are consistent with the weakening of super-exchange interactions between Mn‒Mn ion pairs under compression^59^. In general, the super-exchange strength between transition metal ions is governed by their geometric configuration. When the metal-halide-metal bond angle (Mn‒Cl‒Mn) is 180°, the orbital overlap of electron clouds between adjacent metal ions is maximized, resulting in the strongest super-exchange interaction. Consequently, the lattice compression under pressure reduces the orbital overlap within the Mn–Mn dimer (Fig. 4a), which weakens the super-exchange interaction. This weakened exchange suppresses the spin-canting effect, ultimately leading to the observed blue shift in the PL spectra^39^.

Leveraging the exceptional pressure sensitivity of (C_10_H_20_O_5_Mn)(CH_3_CN)MnCl_4_, we have developed a diving safety alarm system that provides real-time depth monitoring via visible color changes (Fig. 4e). Prior to demonstration the pressure-sensing application, the stability of (C_10_H_20_O_5_Mn)(CH_3_CN)MnCl_4_ was evaluated by thermogravimetric analysis (TGA) (Fig. S10). The operational principle is based on the well-established linear relationship between water pressure and diving depth. The responsive luminescence properties of (C_10_H_20_O_5_Mn)(CH_3_CN)MnCl_4_ enable the indicator to display a distinct color change at specific depth thresholds. When the water pressure is below 2 MPa (corresponding to depths of ≤20 meters), the indicator maintains a steady red-light emission as a safety signal, denoting that the diving depth remains within the safe range. When the water pressure exceeds 5 MPa (≥ 50 meters depth), the lattice compression can induce a blue-shift in emission toward orange-light signal, triggering a multi-level alarm protocol. This includes the indicator transitions to a blinking mode and a pulsed alert by integrated piezoelectric buzzer. When the water pressure reaches 10 MPa pressure or above (≥ 100 meters depth), the lattice’s sustained compression prompts further blue-shift in emission toward a yellow-light signal, along with high-frequency distress signal from piezoelectric alarm. This pressure-responsive luminescent color-changing capability facilitates the real-time water depth monitoring, providing essential fail-safe protection for diving operations.

## Discussion

In summary, we have developed an asymmetric Mn(II) chloride dimer (C_10_H_20_O_5_Mn)(CH_3_CN)MnCl_4_. It features two distinct coordination geometries: a rare 7-coordinated pentagonal bipyramid and a conventional 4-coordinated tetrahedron, which are interconnected by a highly distorted Mn‒Cl‒Mn linkage of 129°. Symmetry breaking induces the spin-canted super-exchange interactions, which give rise to a novel luminescent center characterized by a single-peak red emission at 638 nm with a short lifetime of 0.42 ms. This unique behavior originates from the synergistic interplay between: (i) Mn‒Mn super-exchange coupling and (ii) the relaxation of spin-forbidden *d-d* transitions enabled by spin-canting. The weak ferromagnetic coupling drives a distinct energy-level reconfiguration that separates antibonding orbitals, establishing an electronic band structure distinct from conventional AFM and FM frameworks. Accordingly, it exhibits a stimuli-responsive luminescence: temperature-dependent investigations (5-305 K) reveal a 30 nm blue-shift due to the thermal suppression of spin-canting, while pressure-dependent measurements (0–20 MPa) show a 40 nm blue-shift attributed to the compression-induced reduction in orbital overlap. Capitalizing on its pressure sensitivity, we developed a real-time underwater manometer for depth-resolved surveillance. These findings not only elucidate a new *d-d* transition mechanism in low-symmetry systems but also propel the spin-canting from a magnetic phenomenon to a general design principle for stimuli-responsive optical materials.

## Materials and methods

### Sample preparation

#### Materials

Manganese (Ⅱ) chloride (MnCl_2_, 99%) and 15-crown-5 (C_10_H_20_O_5_, 97%) were purchased from Aladdin Company. Acetonitrile (CH_3_CN, >99.5%) was purchased from Tianjin Kemiou Chemical Reagent Company Limited. All chemical materials were used as received without further purification or additional physical treatment.

#### Synthesis

Add 0.0616 g of MnCl_2_ (0.49 mmol) to 5 mL of CH_3_CN solution and mix rapidly at 70 °C for 5 min. While stirring, 15-crown-5 (50 μL, 0.245 mmol) was added slowly in a suspension. When the solution became clear, the stirring speed was reduced and the reaction temperature was raised to 80 °C. After 30 min of reaction, the stirring was stopped when the solution changes from colorless to pale yellow. The solution was transferred to a temperature-programmed oven and cooled from 80 °C to room temperature at a rate of 2 °C h^−1^, yielding yellow transparent block-shaped single crystals.

### Characterization

The single crystal structure of (C_10_H_20_O_5_Mn)(CH_3_CN)MnCl_4_ was collected at 293(2) K using the XtaLAB AFC12 X-ray four-circle single crystal diffractometer (Rigaku) equipped with Mo-*K*α radiation. Variable-temperature magnetic susceptibility was performed using a Physical Property Measurement System (PPMS). Field-cooled (FC) data were collected under an applied field of 10 kOe from 2 to 300 K. The magnetization curves were measured at 295 K using the vibrating sample magnetometer (VSM) option. Electron spin resonance (EPR) spectra were recorded using a Bruker A300-EPR spectrometer under the following conditions: temperature 298 K, microwave frequency 9.84 GHz, attenuation 10 dB, and modulation amplitude 1 G. Photoluminescence excitation (PLE) spectra and photoluminescence (PL) spectra, and photoluminescence decay curves were performed on a FLS-920 fluorescence spectrophotometer (Edinburgh Instruments Ltd., U.K.) at RT. Temperature-dependent PL spectra were performed on the FLS-920 connected with liquid helium control equipment. Chemical compositions and valences were characterized by a Thermo Scientific K-Alpha X-ray photoelectron spectrometer (XPS). The morphology and elemental composition were obtained by the scanning electron microscope (SEM, JSM6700F) equipped with an Energy Dispersive Spectrometer (EDS). Thermogravimetric analysis (TGA) was performed on SETARAM 131LABSYS equipment under a nitrogen stream with a heating rate of 10 °C min^-1^ from room temperature to 800 °C.

### Calculations

Theoretical simulations were carried out using the Vienna Ab initio Simulation Package (VASP)^60,61^ based on the density functional theory (DFT). The exchange-correlation effect of electrons was described by the Perdew-Burke-Ernzerh (PBE) of the generalized gradient approximation (GGA)^62^. The DFT + U method was employed to treat the strongly correlated 3 *d* electrons of Mn transition metals, with Hubbard parameters set to *U*_ef_ = 4 eV. The plane-wave cutoff energy was set to be 400 eV. The 3 × 3 × 1 k-meshes were used to optimize the structures and calculate the electronic properties. The structures were allowed to relax until the energy on the atoms was less than 1.0 × 10^−5^ eV and all the forces on the atoms were less than 0.1 eV Å^−1^. According to the electronic spin arrangement of Mn^2+^, magnetic structures of four different configurations were built. A default magnetic moment of ±5 *μ*_B_ was set for Mn atoms, and 0 *μ*_B_ was set for other nonmagnetic atoms. The relative energies of the four magnetic configurations were evaluated using spin-polarized DFT + U to identify the magnetic ground state. In addition, the SOC effect was considered to calculate electronic properties.

## Supplementary information

SI

The crystallographic information file (CIF)

checkCIF/PLATON report
