# Supplementary material for: Spin-canted Mn–Mn coupling in symmetry-broken metal chloride dimer with dual-responsive luminescence and sensing
Source: Light Sci Appl. 2026 Jan 28;15:90. doi: 10.1038/s41377-025-02154-9 (PMC12852691; doi:10.1038/s41377-025-02154-9)
Supplement: Supplementary file 1 — SI [file 41377_2025_2154_MOESM1_ESM.docx]

**[Supplementary Information](https://static-content.springer.com/esm/art%3A10.1038%2Fs41467-022-35366-3/MediaObjects/41467_2022_35366_MOESM1_ESM.pdf)**

**Spin-Canted** **Mn–Mn Coupling in** **Symmetry-Broken** **Metal Chloride Dimer** **with Dual-Responsive** **Luminescence and Sensing**

Guojun Zhou^1, #, *^, Pei Wang^1, #^, Qiqiong Ren^1, #^, Nan Zhang^1^, Jin Lv^1^, Yilin Mao^1^, Jianwei Qiao^2, *^, Xian-Ming Zhang^1, 3, *^

^1^Key Laboratory of Magnetic Molecules and Magnetic Information Material, Ministry of Education, School of Chemistry and Chemistry Engineering, Shanxi Normal University, Taiyuan 030031, China

^2^College of Physics and Optoelectronics, Taiyuan University of Technology, Taiyuan 030024, China

^3^Department of Applied Chemistry, Yuncheng University, Yuncheng, Shanxi 044000, China

^#^These three authors as co-first authors contributed equally to this work.

^*^E-mail: [zhougj@sxnu.edu.cn](mailto:mailtozhougj@sxnu.edu.cn) (G. J. Z.); [qiaojianwei@tyut.edu.cn](mailto:qiaojianwei@tyut.edu.cn) (Q. J. W.); [zhangxianming@tyut.edu.cn](mailto:zhangxianming@tyut.edu.cn) (X. M. Z.)


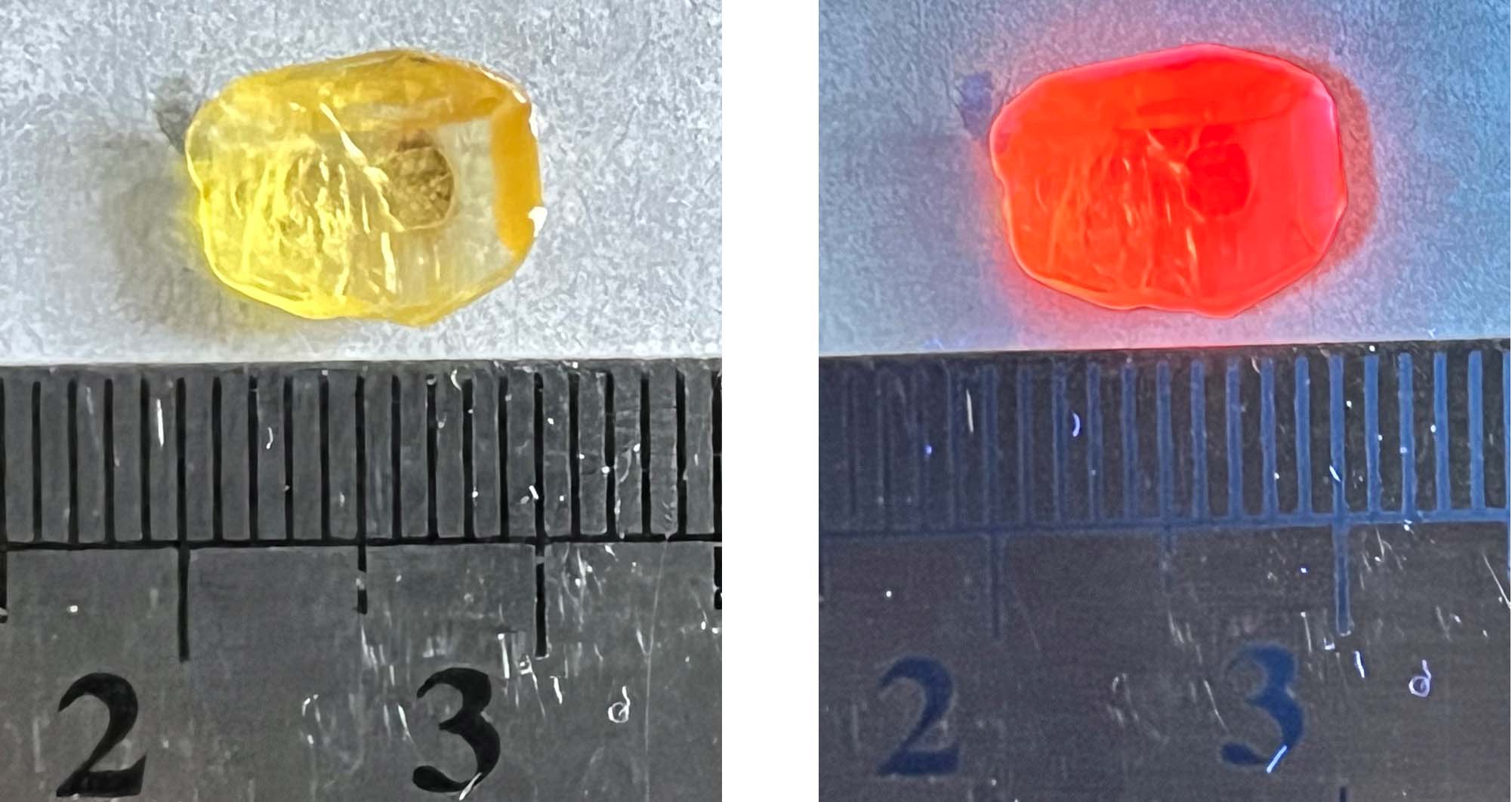


**Fig. S1** Centimeter-scale single crystals of (C_10_H_20_O_5_Mn)(CH_3_CN)MnCl_4_ under natural light and UV lamp.


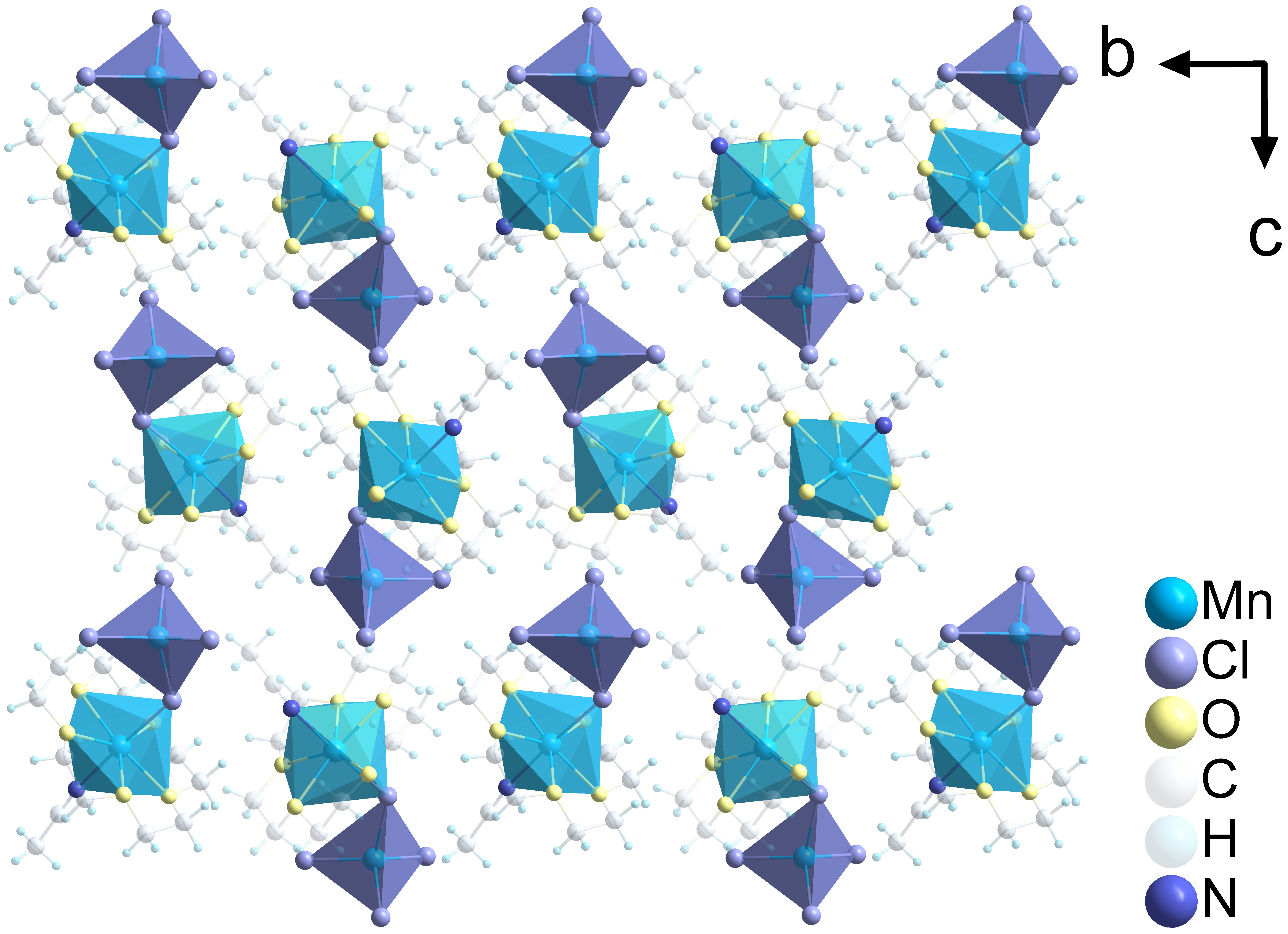


**Fig. S2** Packing views of (C_10_H_20_O_5_Mn)(CH_3_CN)MnCl_4_ unit cell along *a* axis.


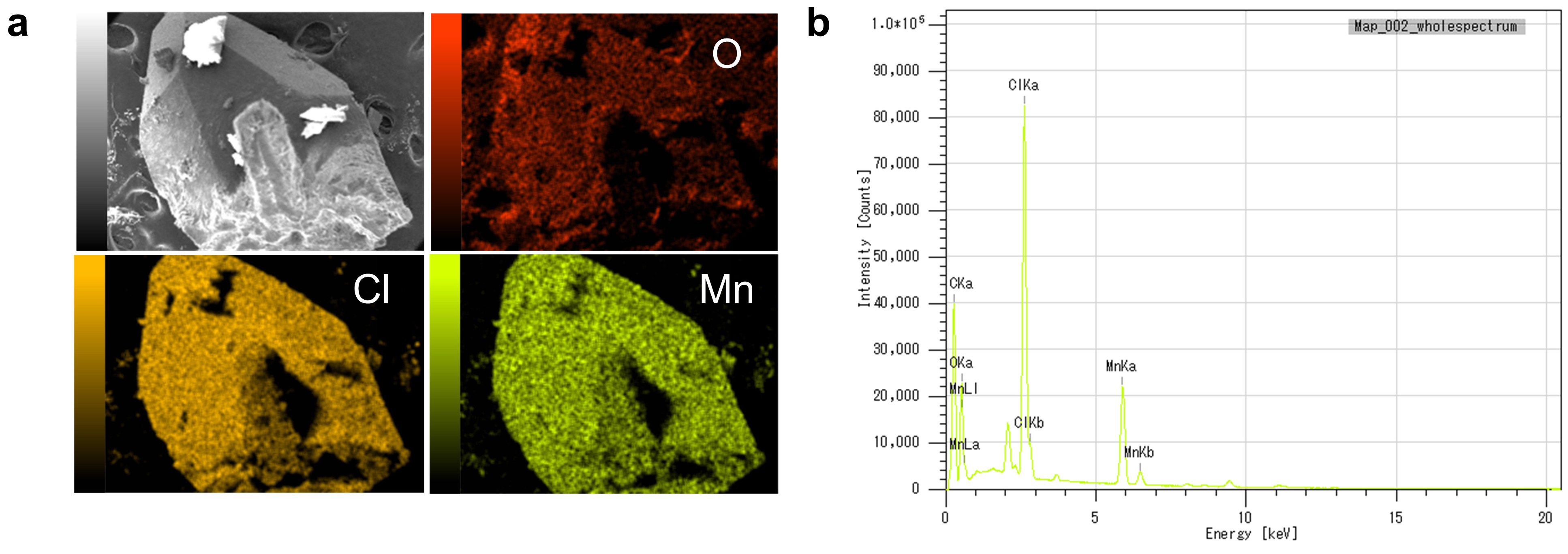


**Fig. S3** **a)** Scanning electron microscopy (SEM) and elemental mappings of Mn, O, Cl **b)** Energy dispersive spectrometer EDS elemental mappings of (C_10_H_20_O_5_Mn)(CH_3_CN)MnCl_4_.


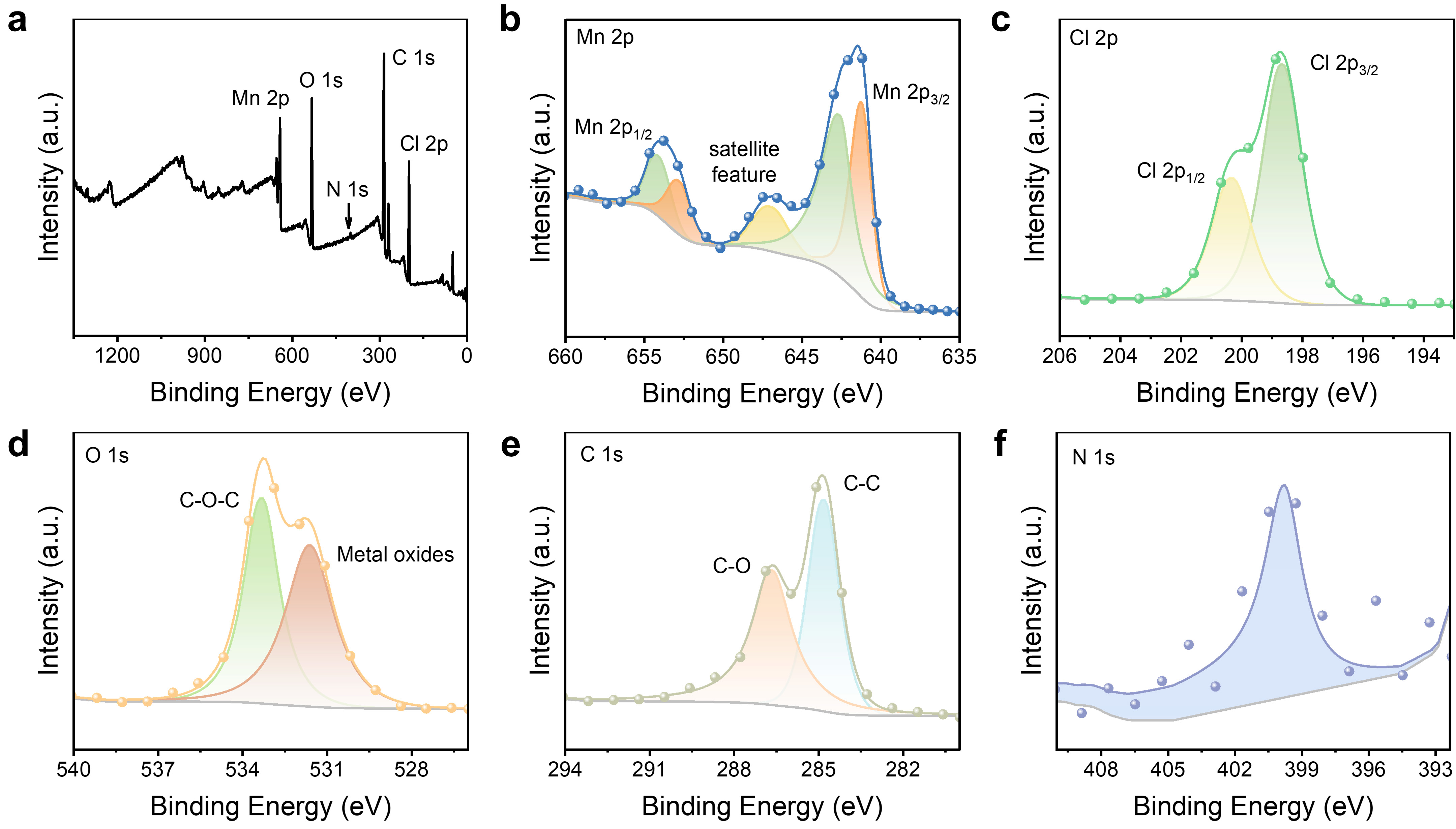


**Fig. S4** XPS spectra for **a)** survey scan and high-resolution XPS spectra of **b)** Mn 2*p*; **c**) Cl 2*p*; **d)** O 1*s*; **e)** C 1*s*; **f)** N 1*s* in (C_10_H_20_O_5_Mn)(CH_3_CN)MnCl_4_.


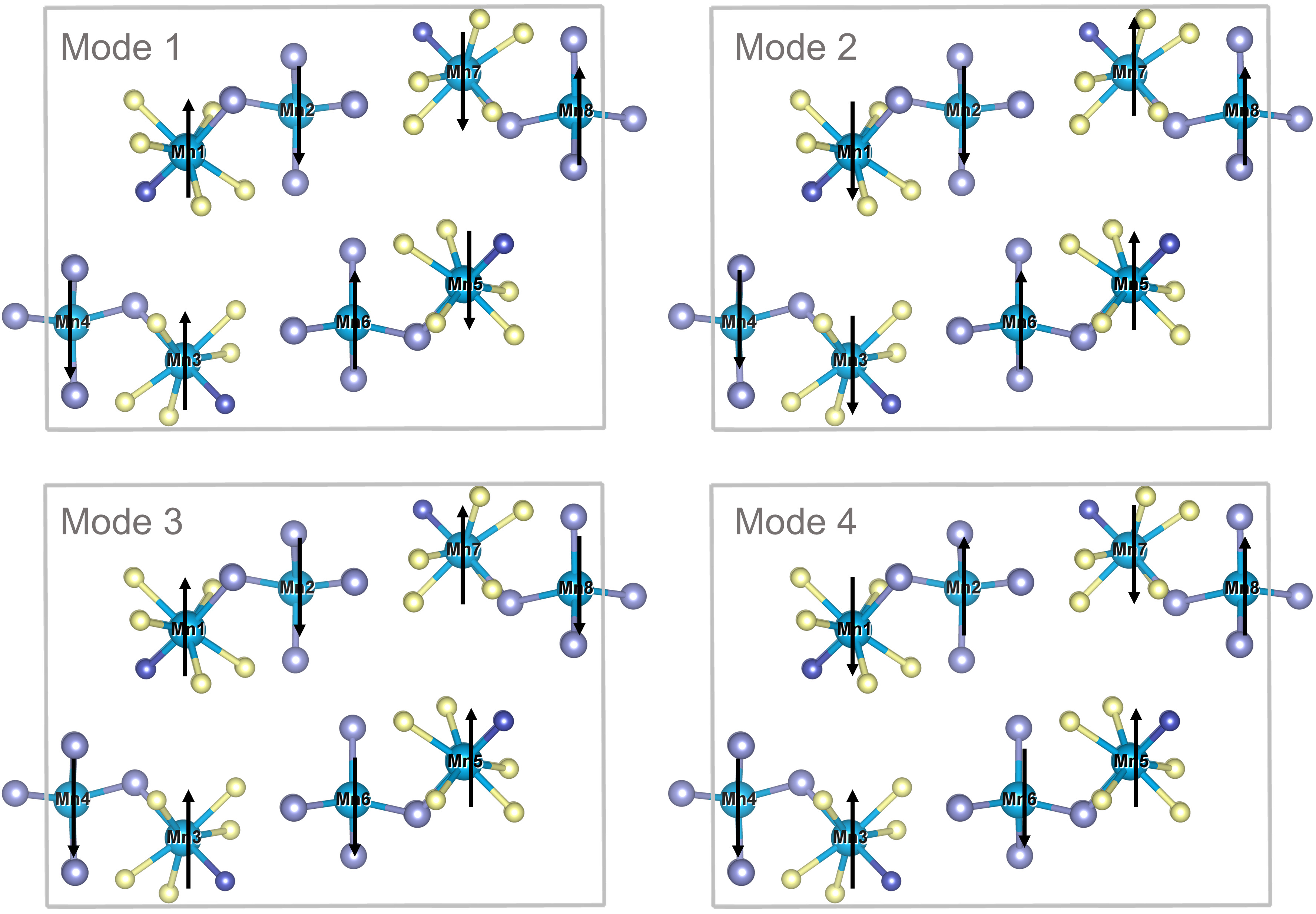


**Fig. S5** Magnetic structures including three different AFM magnetic models (Mode1, 3, 4) and one FM magnetic model (Mode 2).


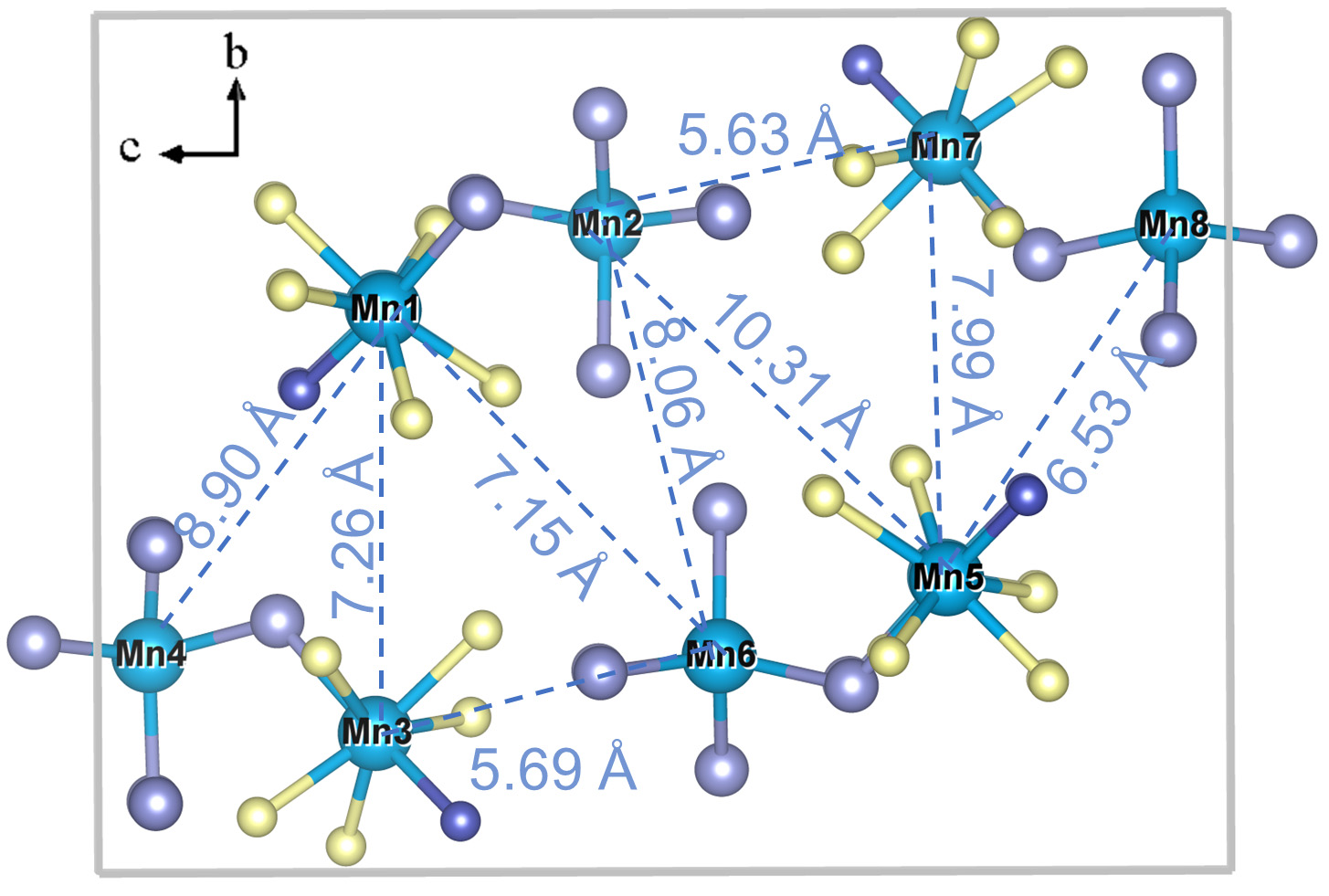


**Fig. S6.** The Mn···Mn distances between different dimers.


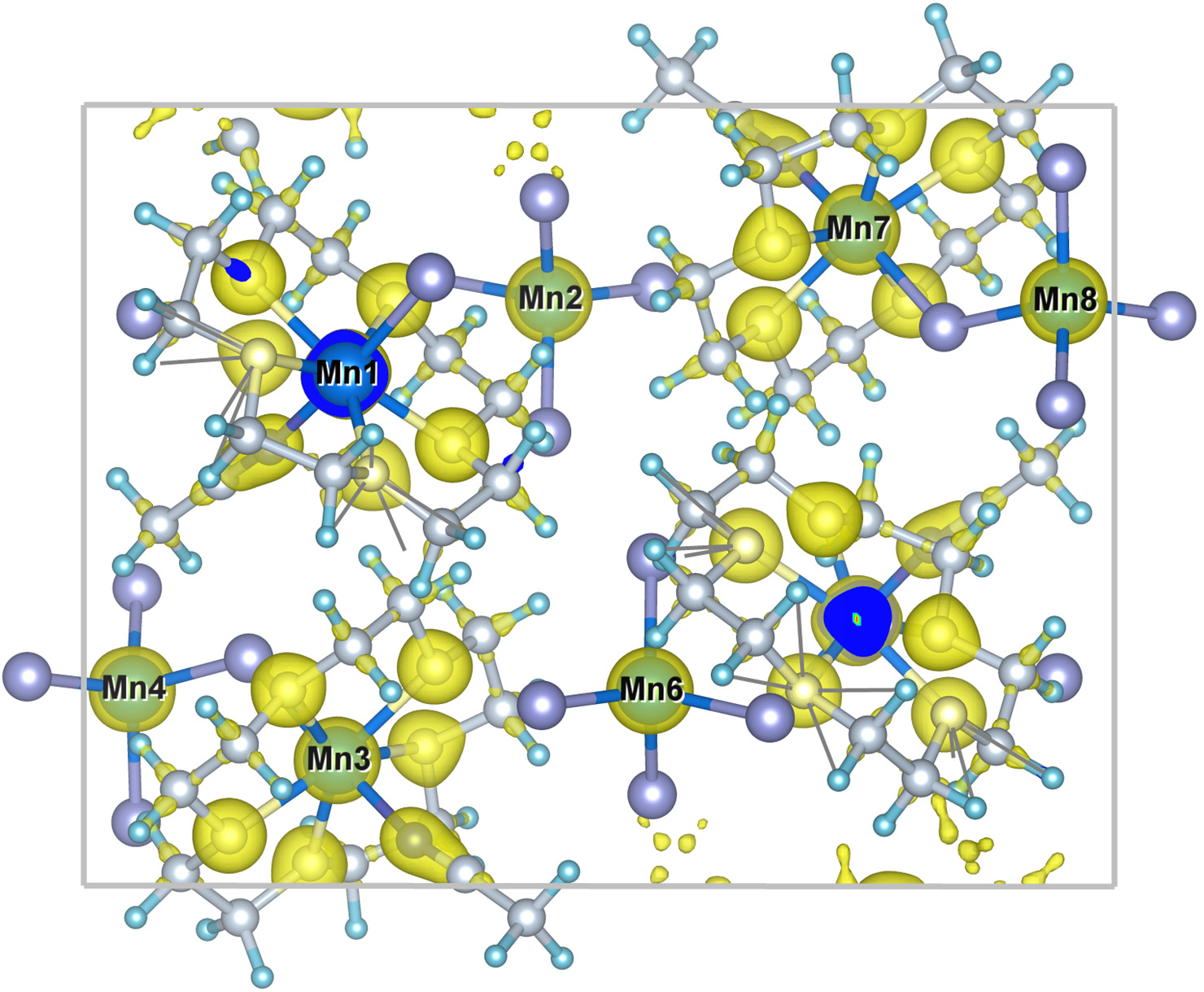


**Fig. S****7** The plot depicting the charge density distribution of (C_10_H_20_O_5_Mn)(CH_3_CN)MnCl_4_. The yellow and cyan areas signify the charge accumulation and depletion.


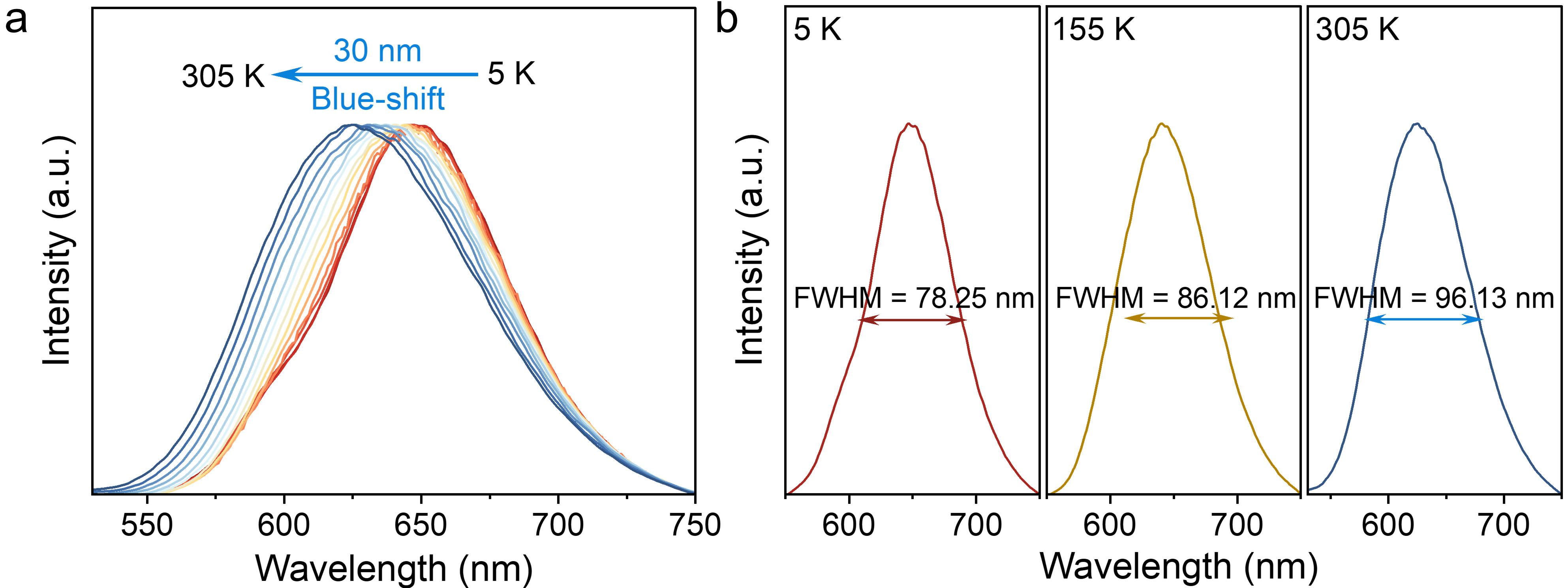


**Fig. S8** Temperature-dependent PL spectra of (C_10_H_20_O_5_Mn)(CH_3_CN)MnCl_4_ from 5 to 305 K. **a)** Temperature-induced blue-shift from 668 nm to 638 nm in spin-canted Mn^2+^ dimer. **b)** Emission spectra from 5 to 305 K with a broadening of the FWHM.


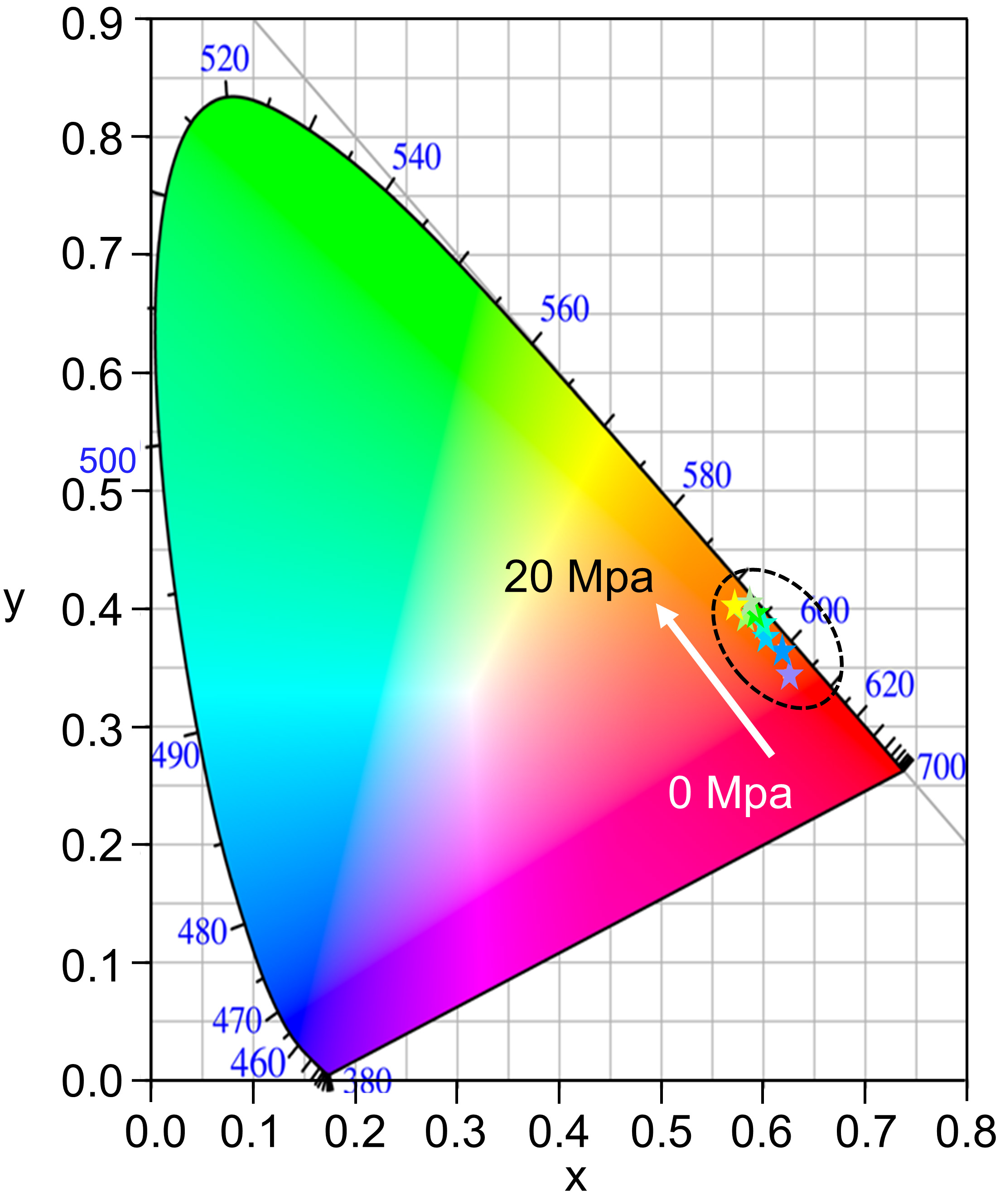


**Fig. S9** The pressures-dependent CIE plots in the range of 0-20 MPa.


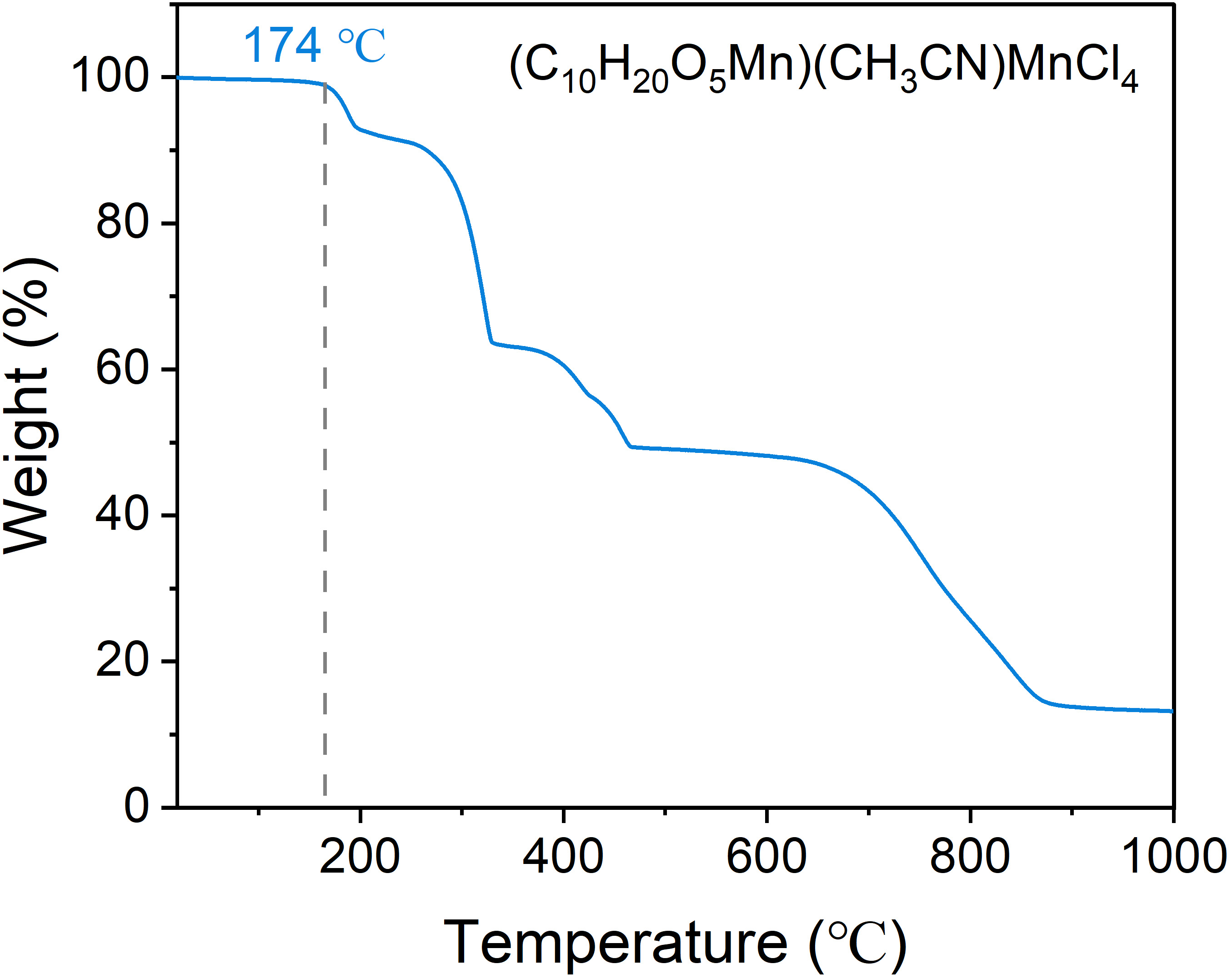


**Fig. S10** The TGA analysis spectrum of (C_10_H_20_O_5_Mn)(CH_3_CN)MnCl_4_.

**Table S1** Main parameters of processing and refinement of the (C_10_H_20_O_5_Mn)(CH_3_CN)MnCl_4_ at room temperature.

| **Chemical formula** | **C_12_H_23_O_5_Cl_4_Mn_2_N** |
| --- | --- |
| Molecular weight | 512.99 |
| Temperature (K) | 293(2) |
| Space Group, *Z* | *P*2_1_/*n*, 4 |
| *a* (Å) | 9.2163(18) |
| *b* (Å) | 13.278(3) |
| *c* (Å) | 17.365(4) |
| *α*(°) | 90 |
| *β* (°) | 95.41(3) |
| *γ* (°) | 90 |
| *V* (Å^3^) | 2115.6(7) |
| ρ_calc_(g/cm^3^) | 1.611 |
| μ (mm^-1^) | 1.718 |
| Reflections measured | 31580 |
| Reflections independent | 4337 |
| Reflections with *F* > 4σ(*F*) | 3585 |
| 2θ_max_ (°) | 52.896 |
| *h*, *k*, *l* - limits | -11 ≤ *h* ≤ 11; -16 ≤ *k* ≤ 16; -21 ≤ *l* ≤ 21 |
| *R*_int_ | 0.0585 |
| *Refinement results* |  |
| The weighed refinement of *F*^2^ | *w* = 1/[*σ*^2^(*F*_o_^2^)+(0.0532*P*)^2^+0.0369*P*]  where *P* = max(*F*_o_^2^+2*F*_c_^2^)/3 |
| Number of refinement parameters | 218 |
| *R*_1_[*F*_o_ > 4σ(*F*_o_)] | 0.0324 |
| *wR2* | 0.0811 |
| *Goof* | 1.130 |
| ∆*ρ*_max_(e/Å^3^) | 0.729 |
| ∆*ρ*_min_(e/Å^3^) | -0.765 |
| (∆/*σ*)_max_ | <0.001 |
| Extinction coefficient (SHELXL 2014/7) | 0.0150(8) |

**Table S2** Fractional atomic coordinates and isotropic or equivalent isotropic displacement parameters (Å^2^).

| **Atom** | ***x*** | ***y*** | ***z*** | ***U*_iso_**/U*_eq_** | ***Occ.*** |
| --- | --- | --- | --- | --- | --- |
| Mn1 | 0.46654(3) | 0.66066(2) | 0.74523(2) | 0.03225(12) | 1 |
| Mn2 | 0.72944(4) | 0.75117(2) | 0.55115(2) | 0.03502(12) | 1 |
| Cl1 | 0.59700(7) | 0.78352(5) | 0.66373(3) | 0.04804(18) | 1 |
| Cl2 | 0.83632(7) | 0.59013(5) | 0.55041(4) | 0.04768(17) | 1 |
| Cl3 | 0.91484(7) | 0.87180(5) | 0.55608(4) | 0.05249(18) | 1 |
| Cl4 | 0.54649(8) | 0.76611(6) | 0.44780(4) | 0.0600(2) | 1 |
| O1 | 0.35917(17) | 0.56807(12) | 0.64603(8) | 0.0405(4) | 1 |
| O2 | 0.26032(18) | 0.73918(12) | 0.70301(9) | 0.0432(4) | 1 |
| O3 | 0.42099(18) | 0.77479(12) | 0.83388(9) | 0.0461(4) | 1 |
| O4 | 0.66303(19) | 0.66979(15) | 0.83129(9) | 0.0528(5) | 1 |
| O5 | 0.61244(19) | 0.53916(14) | 0.72042(10) | 0.0559(5) | 1 |
| C1 | 0.2489(3) | 0.6235(2) | 0.59997(14) | 0.0553(7) | 1 |
| C2 | 0.1604(3) | 0.6768(2) | 0.65459(16) | 0.0556(7) | 1 |
| C3 | 0.1945(3) | 0.7950(2) | 0.76103(15) | 0.0540(7) | 1 |
| C4 | 0.3156(3) | 0.8487(2) | 0.80817(16) | 0.0596(8) | 1 |
| C5 | 0.5464(3) | 0.8098(3) | 0.88059(16) | 0.0651(8) | 1 |
| C6 | 0.6395(3) | 0.7209(3) | 0.90093(15) | 0.0686(9) | 1 |
| C7 | 0.7454(3) | 0.5803(3) | 0.83854(18) | 0.0743(9) | 1 |
| C8 | 0.7499(3) | 0.5329(2) | 0.76267(18) | 0.0704(8) | 1 |
| C9 | 0.5757(3) | 0.47153(18) | 0.65751(15) | 0.0515(6) | 1 |
| C10 | 0.4650(3) | 0.52177(19) | 0.60211(14) | 0.0484(6) | 1 |
| C11 | 0.2810(3) | 0.51932(18) | 0.86542(13) | 0.0425(5) | 1 |
| C12 | 0.2040(3) | 0.4657(2) | 0.92163(14) | 0.0583(7) | 1 |
| N1 | 0.3402(2) | 0.56047(16) | 0.82105(11) | 0.0500(5) | 1 |
| H2A | 0.187985 | 0.578032 | 0.567283 | 0.066* | 1 |
| H2B | 0.293269 | 0.671480 | 0.567236 | 0.066* | 1 |
| H3A | 0.086419 | 0.718038 | 0.626408 | 0.067* | 1 |
| H3B | 0.112637 | 0.628752 | 0.685854 | 0.067* | 1 |
| H5A | 0.144064 | 0.750022 | 0.793505 | 0.065* | 1 |
| H5B | 0.124840 | 0.843107 | 0.737210 | 0.065* | 1 |
| H6A | 0.358856 | 0.899136 | 0.776979 | 0.072* | 1 |
| H6B | 0.278676 | 0.881965 | 0.852075 | 0.072* | 1 |
| H8A | 0.517799 | 0.841757 | 0.927067 | 0.078* | 1 |
| H8B | 0.599020 | 0.858576 | 0.852286 | 0.078* | 1 |
| H9A | 0.731844 | 0.742130 | 0.927358 | 0.082* | 1 |
| H9B | 0.591730 | 0.676518 | 0.935027 | 0.082* | 1 |
| H11A | 0.702123 | 0.534355 | 0.873249 | 0.089* | 1 |
| H11B | 0.843781 | 0.595472 | 0.860369 | 0.089* | 1 |
| H12A | 0.822048 | 0.566296 | 0.734448 | 0.084* | 1 |
| H12B | 0.777943 | 0.462750 | 0.769294 | 0.084* | 1 |
| H14A | 0.536395 | 0.409485 | 0.676442 | 0.062* | 1 |
| H14B | 0.661957 | 0.455392 | 0.631946 | 0.062* | 1 |
| H15A | 0.511391 | 0.572222 | 0.572301 | 0.058* | 1 |
| H15B | 0.418414 | 0.472620 | 0.566598 | 0.058* | 1 |
| H18A | 0.268599 | 0.418139 | 0.948745 | 0.070* | 1 |
| H18B | 0.122476 | 0.430566 | 0.895617 | 0.070* | 1 |
| H18C | 0.169803 | 0.512703 | 0.957819 | 0.070* | 1 |

**Table S3** The main bond lengths (Å) of (C_10_H_20_O_5_Mn)(CH_3_CN)MnCl_4_.

| **Atom‒Atom** | **Length/Å** | **Atom‒Atom** | **Length/Å** |
| --- | --- | --- | --- |
| Mn1‒O1 | 2.2664(16) | C3‒C4 | 1.501(4) |
| Mn1‒O2 | 2.2301(17) | C3‒O2 | 1.432(3) |
| Mn1‒O3 | 2.2283(15) | C4‒O3 | 1.423(3) |
| Mn1‒O4 | 2.2399(18) | C5‒C6 | 1.482(5) |
| Mn1‒O5 | 2.1690(17) | C5‒O3 | 1.426(3) |
| Mn1‒N1 | 2.2690(2) | C6‒O4 | 1.421(3) |
| Mn1‒Cl1 | 2.5359(8) | C7‒C8 | 1.465(4) |
| Mn2‒Cl1 | 2.4386(9) | C7‒O4 | 1.409(4) |
| Mn2‒Cl2 | 2.3548(8) | C8‒O5 | 1.405(3) |
| Mn2‒Cl3 | 2.3378(8) | C9‒C10 | 1.492(4) |
| Mn2‒Cl4 | 2.3519(10) | C9‒O5 | 1.430(3) |
| C1‒C2 | 1.487(4) | C10‒O1 | 1.432(3) |
| C1‒O1 | 1.435(3) | C11‒N1 | 1.126(3) |
| C2‒O2 | 1.448(3) | C11‒C12 | 1.447(3) |

**Table S4** Bond Angles for (C_10_H_20_O_5_Mn)(CH_3_CN)MnCl_4_.

| **Atom‒Atom‒Atom** | **Angle/°** | **Atom‒Atom‒Atom** | **Angle/°** |
| --- | --- | --- | --- |
| O1‒Mn1‒Cl1 | 96.94(4) | C1‒O1‒Mn1 | 112.42(14) |
| O1‒Mn1‒N1 | 84.93(7) | C10‒O1‒Mn1 | 111.53(13) |
| O2‒Mn1‒Cl1 | 87.14(5) | C10‒O1‒C1 | 113.90(18) |
| O2‒Mn1‒O1 | 72.91(6) | O1‒C1‒C2 | 106.88(19) |
| O2‒Mn1‒O4 | 143.54(7) | O2‒C2‒C1 | 106.6(2) |
| O2‒Mn1‒N1 | 89.81(7) | C2‒O2‒Mn1 | 113.52(14) |
| O3‒Mn1‒Cl1 | 94.48(5) | C3‒O2‒Mn1 | 114.55(14) |
| O3‒Mn1‒O1 | 142.83(6) | C3‒O2‒C2 | 114.6(2) |
| O3‒Mn1‒O2 | 72.48(6) | O2‒C3‒C4 | 106.7(2) |
| O3‒Mn1‒O4 | 72.26(7) | O3‒C4‒C3 | 107.1(2) |
| O3‒Mn1‒N1 | 81.81(7) | C4‒O3‒Mn1 | 114.84(14) |
| O4‒Mn1‒Cl1 | 86.64(5) | C4‒O3‒C5 | 116.5(2) |
| O4‒Mn1‒O1 | 143.53(6) | C5‒O3‒Mn1 | 114.79(15) |
| O4‒Mn1‒N1 | 94.11(7) | O3‒C5‒C6 | 107.2(2) |
| O5‒Mn1‒Cl1 | 91.85(6) | O4‒C6‒C5 | 108.0(2) |
| O5‒Mn1‒O1 | 71.34(6) | C6‒O4‒Mn1 | 114.53(15) |
| O5‒Mn1‒O2 | 143.84(6) | C7‒O4‒Mn1 | 114.15(16) |
| O5‒Mn1‒O3 | 143.49(7) | C7‒O4‒C6 | 116.8(2) |
| O5‒Mn1‒O4 | 72.28(7) | O4‒C7‒C8 | 110.1(2) |
| O5‒Mn1‒N1 | 92.34(8) | O5‒C8‒C7 | 110.1(2) |
| N1‒Mn1‒Cl1 | 175.78(6) | C8‒O5‒Mn1 | 119.07(16) |
| Cl2‒Mn2‒Cl1 | 113.93(3) | C8‒O5‒C9 | 119.9(2) |
| Cl3‒Mn2‒Cl1 | 105.62(3) | C9‒O5‒Mn1 | 120.83(15) |
| Cl3‒Mn2‒Cl2 | 108.52(3) | O5‒C9‒C10 | 108.03(19) |
| Cl3‒Mn2‒Cl4 | 116.04(3) | O1‒C10‒C9 | 107.90(19) |
| Cl4‒Mn2‒Cl1 | 102.70(3) | C11‒N1‒Mn1 | 171.6(2) |
| Cl4‒Mn2‒Cl2 | 110.03(3) | N1‒C11‒C12 | 179.3(3) |
| Mn2‒Cl1‒Mn1 | 129.32(3) |  |  |

**Table S5** Hydrogen-bond geometry in the (C_10_H_20_O_5_Mn)(CH_3_CN)MnCl_4_ structure (Å, °).

| ***D*‒H···*A*** | ***D*‒H** | **H···*A*** | ***D*···*A*** | ***D*‒H···*A*** |
| --- | --- | --- | --- | --- |
| C2‒H2*A*···Cl3^i^ | 0.97 | 2.79 | 3.745 (3) | 167.2 |
| C3‒H3*A*···Cl4^ii^ | 0.97 | 2.91 | 3.725 (3) | 141.9 |
| C5‒H5*A*···Cl2^ii^ | 0.97 | 2.98 | 3.910 (3) | 161.0 |
| C6‒H6*A*···Cl4^iii^ | 0.97 | 2.89 | 3.766 (3) | 150.8 |
| C6‒H6*B*···Cl3^ii^ | 0.97 | 2.85 | 3.757 (3) | 155.6 |
| C7‒H7*A*···Cl3^iv^ | 0.97 | 2.75 | 3.703 (4) | 166.4 |
| C7‒H7*B*···Cl4^iii^ | 0.97 | 2.94 | 3.802 (3) | 148.9 |
| C8‒H8*B*···Cl1^iv^ | 0.97 | 2.84 | 3.774 (3) | 160.8 |
| C9‒H9*B*···Cl2 | 0.97 | 2.87 | 3.543 (3) | 127.5 |
| C12‒H12*B*···Cl1^v^ | 0.96 | 2.93 | 3.869 (3) | 166.8 |

Symmetry codes: (i) *x*-1, *y*, *z*; (ii) *x*-1/2, -*y*+3/2, *z*+1/2; (iii) *x*+1/2, -*y*+3/2, *z*+1/2; (iv) -*x*+3/2, *y*-1/2, -*z*+3/2; (v) -*x*+1/2, *y*-1/2, -*z*+3/2.

**Table S6** Total energy for various hypothetic models.

| **Mode** | **Total Energy (eV)** |
| --- | --- |
| 1 | -1066.01607 |
| 2 | -1065.96361 |
| 3 | -1066.01616 |
| 4 | -1066.01605 |

**Table S7** Comparison of fluorescent lifetime parameters of 0D hybrid Mn(II) chloride.

|  | **Single crystal** | **Fluorescence lifetime** | **References** |
| --- | --- | --- | --- |
| 1 | TAMn_2_Cl_5_⋅2H_2_O | 1.14 ms | 1 |
| 2 | (*S*‑C_3_H_10_ON)MnCl_3_ | 1.02 ms | 2 |
| 3 | (C_4_NOH_10_)_5_Mn_2_Cl_9_·C_2_H_5_OH | 4.83 ms | 3 |
|  | (ABI)_4_MnCl_6_ | 13.19 ms | 4 |
| 4 | Mn-2DMAP | 1.21 ms | 5 |
| 5 | Mn-4DMAP | 1.79 ms | 5 |
| 6 | (C_4_NOH_10_)_2_MnCl4 | 3.36 ms | 3 |
| 7 | (C_13_H_26_N)_2_MnCl_4_ | 3.829 ms | 6 |
| 8 | (C_7_H_18_N)_2_MnCl_4_ | 4.206 ms | 6 |
| 9 | (C_8_H_20_N)_2_MnCl_4_ | 3.42 ms | 7 |
| 10 | (Ph4P)_2_MnCl_4_ | 3.9 ms | 8 |
| 11 | (C_9_H_12_N_2_)_2_MnCl_4_ | 2.094 ms | 9 |
| 12 | (C_5_H_13_N_3_)_2_MnCl_4_ | 3.54 ms | 10 |
| 13 | (C_24_H_20_P)_2_MnCl_4_ | 2.46 ms | 11 |
| 14 | [DMAEMP]MnCl_4_ | 3.26 ms | 12 |
| 15 | [MMPrIm]MnCl_4_ | 3.527 ms | 12 |
| 16 | [EP]MnCl_4_ | 1.40 ms | 12 |
| 17 | (KC)_2_MnCl_4_ | 2.79 ms | 13 |

**Table S8** Relationship between line width (ΔH) and pressure variation based on EPR.

| **Pressure (MPa)** | **ΔH (G)** |
| --- | --- |
| 0 | 406.17 |
| 2 | 411.76 |
| 3 | 442.09 |
| 4 | 450.07 |
| 5 | 456.46 |
| 10 | 459.64 |
| 15 | 460.45 |
| 20 | 458.84 |

**References**

1. Cong, L., et al. Manganese halides with timely and delayed reversible thermal fluorescence quenching for dual-secure information encryption and anti-counterfeiting. *Chemical Engineering Journal* **505**, 159672 (2025).

2. Panda, D. P., et al. Negative thermal quenching and self-trapped exciton emission in (R/S-C_3_H_10_ON)MnCl_3_. *Chemistry of Materials* **36**, 5698-5708 (2024).

3. Sun, M. E., et al. Thermoinduced structural-transformation and thermochromic luminescence in organic manganese chloride crystals. *Chemical Science* **10**, 3836-3839 (2019).

4. Wang, Y. L., et al. Flexible 3D printed fibers of novel manganese halides with superior acid/alkali resistance for X-ray imaging and radiation warning. *Chemical Engineering Journal* **515**, 163495 (2025).

5. Zhou, B., et al. Dynamic magneto-optical response and reversible humidity-sensitive luminescence tuned by intermolecular interactions in manganese halides. *Advanced Functional Materials* **35**, 2503326 (2025).

6. Wang, N., et al. Efficient narrow-band green light-emitting hybrid halides for wide color gamut display. *ACS Applied Electronic Materials* **4**, 4068-4076 (2022).

7. Chang, T., et al. Temperature-dependent reversible optical properties of Mn-based organic–inorganic hybrid (C_8_H_20_N)_2_MnCl_4_ metal halides. *ACS Applied Materials & Interfaces* **15**, 5487-5494 (2023).

8. Ben‐Akacha, A., et al. Mechanochemical synthesis of zero dimensional organic‐inorganic metal halide hybrids. *ChemPhotoChem* **4**, 1–5 (2020).

9. Cong, L., et al. Reversible thermodynamic phase-transition in zero-dimensional manganese halides enables dual-secure optical information anti-counterfeiting and encryption. *Laser & Photonics Reviews* **19**, e00761 (2025).

10. Cheng, X. H., et al. Centimeter‐sized single crystals of tetrahedral manganese (Ⅱ) halide hybrids for wide‐color gamut backlighting displays. *Small*, 2307216 (2023).

11. Zhou, G. J., et al. Manipulation of Cl/Br transmutation in zero-dimensional Mn^2+^-based metal halides toward tunable photoluminescence and thermal quenching behaviors. *Journal of Materials Chemistry C* **9**, 2047-2053 (2021).

12. Pan, H. M., et al. Enhancement of the photoluminescence efficiency of hybrid manganese halides through rational structural design. *Chemical Communications* **57**, 6907-6910 (2021).

13. Zhao, J., et al. Circularly polarized luminescence from achiral single crystals of hybrid manganese halides. *Journal of the American Chemical Society* **141**, 15755-15760 (2019).
